# Managing Obesity in Young Children: A Multiple Methods Study Assessing Feasibility, Acceptability, and Implementation of a Multicomponent, Family-Based Intervention

**DOI:** 10.1089/chi.2021.0221

**Published:** 2022-08-29

**Authors:** Laura M. Kinlin, Stephan M. Oreskovich, Raluca Dubrowski, Geoff D.C. Ball, Melanie Barwick, Elizabeth Dettmer, Jess Haines, Jill Hamilton, Theresa H.M. Kim, Marie Klaassen, Paola Luca, Jonathon L. Maguire, Myla E. Moretti, Elaine Stasiulis, Alene Toulany, Catherine S. Birken

**Affiliations:** ^1^ Division of Paediatric Medicine, The Hospital for Sick Children, Toronto, Ontario, Canada.; ^2^ Department of Paediatrics, Temerty Faculty of Medicine, University of Toronto, Toronto, Ontario, Canada.; ^3^ Child Health Evaluative Sciences, SickKids Research Institute, Toronto, Ontario, Canada.; ^4^ Division of Endocrinology, The Hospital for Sick Children, Toronto, Ontario, Canada.; ^5^ Department of Pediatrics, University of Alberta, Edmonton, Alberta, Canada.; ^6^ Department of Psychiatry, Temerty Faculty of Medicine, University of Toronto, Toronto, Ontario, Canada.; ^7^ Institute of Health Policy, Management and Evaluation, Dalla Lana School of Public Health, University of Toronto, Toronto, Ontario, Canada.; ^8^ Department of Psychology, The Hospital for Sick Children, Toronto, Ontario, Canada.; ^9^ Department of Family Relations and Applied Nutrition, University of Guelph, Guelph, Ontario, Canada.; ^10^ Department of Nutritional Sciences, Temerty Faculty of Medicine, University of Toronto, Toronto, Ontario, Canada.; ^11^ Toronto Public Health, Toronto, Ontario, Canada.; ^12^ Section of Pediatric Endocrinology, Department of Pediatrics, Alberta Children's Hospital, University of Calgary, Calgary, Alberta, Canada.; ^13^ Department of Pediatrics, St. Michael's Hospital, Unity Health Toronto, Toronto, Ontario, Canada.; ^14^ Li Ka Shing Knowledge Institute, St. Michael's Hospital, Unity Health Toronto, Toronto, Ontario, Canada.; ^15^ Clinical Trials Unit, Ontario Child Health Support Unit, The Hospital for Sick Children, Toronto, Ontario, Canada.; ^16^ Institute of Medical Science, Temerty Faculty of Medicine, University of Toronto, Toronto, Ontario, Canada.; ^17^ Division of Adolescent Medicine, The Hospital for Sick Children, Toronto, Ontario, Canada.

**Keywords:** acceptability, feasibility, implementation, pediatric obesity, pilot RCT

## Abstract

**Background:**

We developed a multicomponent, family-based intervention for young children with obesity consisting of parent group sessions, home nursing visits, and multidisciplinary clinical encounters. Our objective was to assess intervention feasibility, acceptability, and implementation.

**Methods:**

From 2017 to 2020, we conducted a multiple methods study in the obesity management clinic at a tertiary children's hospital (Toronto, Canada). We included 1–6 year olds with a body mass index ≥97th percentile and their parents; we also included health care providers (HCPs) who delivered the intervention. To assess feasibility, we performed a pilot randomized controlled trial (RCT) comparing the intervention to usual care. To explore acceptability, we conducted parent focus groups. To explore implementation, we examined contextual factors with HCPs using the Consolidated Framework for Implementation Research.

**Results:**

There was a high level of ineligibility (*n* = 34/61) for the pilot RCT. Over 21 months, 11 parent-child dyads were recruited; of 6 randomized to the intervention, 3 did not participate in group sessions or home visits. In focus groups, themes identified by parents (*n* = 8) related to information provided at referral; fit between the intervention and patient needs; parental gains from participating in the intervention; and feasibility of group sessions. HCPs (*n* = 10) identified contextual factors that were positively and negatively associated with intervention implementation.

**Conclusions:**

We encountered challenges related to intervention feasibility, acceptability, and implementation. Lessons learned from this study will inform the next iteration of our intervention and are relevant to intervention development and implementation for young children with obesity.

Clinical Trial Registration number: NCT03219658.

## Introduction

Obesity in childhood often persists into adulthood.^1^ In a large, longitudinal analysis, 84% of children with obesity had obesity as adults, and all children with severe obesity had obesity as adults.^2^ Innovative and evidence-based interventions for managing obesity are required to minimize health consequences, optimize care, reduce system-related costs, and enhance family well-being.

Early childhood represents an opportune time for obesity intervention, for numerous reasons. First, obesity, even early in life, has been linked to important effects (*e.g.*, mental health service utilization).^3^ Second, early-life behaviors and patterns (*e.g.*, low physical activity, short sleep duration) are well-established correlates in the development and persistence of obesity.^4^ Third, behavioral and lifestyle modifications appear to be more successful when implemented at a younger age.^5,6^

In children younger than 12 years of age, family-based interventions addressing nutrition, physical activity, sedentary activity, and sleep represent best practice^7^; yet, evidence of effectiveness is sparse for children younger than 6 years of age.^8^ Limited studies suggest that multicomponent interventions are effective in this age group.^8^ Parent-focused group sessions and home visits appear to be promising intervention components for young children with obesity. Parent-focused group sessions recognize caregivers as “agents of change”^9^ and have shown evidence of effectiveness.^10,11^ Home visits have the potential to increase access and applicability of care for families.^12,13^ In the literature, a single intervention has incorporated both parent-focused group sessions and home visits for young children with obesity; that intervention was effective in reducing body mass index (BMI) compared to usual clinical care.^14–16^

Based on emerging evidence and expertise of specialists in obesity management, we developed a family-based intervention for young children, incorporating parent group sessions and home nursing visits—the SickKids Team Obesity Management Early Years (STOMP-EY) intervention. We planned and conducted a pilot randomized controlled trial (RCT) to inform a definitive assessment of clinical effectiveness, but encountered significant challenges with recruitment and participation. These challenges highlighted the need to carefully consider intervention implementation.

Ensuring acceptability and feasibility, and assessing contextual factors that impact implementation in real-world settings, is critical for effective implementation.^17^ The purpose of this study was to assess feasibility, acceptability, and contextual factors affecting implementation of the STOMP-EY intervention.

## Methods

### Design and Setting

Between October 2017 and March 2020, we studied STOMP-EY using multiple methods: (1) a pilot RCT, to assess feasibility, (2) parent focus groups, to explore acceptability (qualitative), and (3) health care provider (HCP) interviews, to assess contextual factors impacting implementation (qualitative). This was a single-center study conducted in the SickKids Team Obesity Management Program (STOMP), a pediatric obesity management clinic within a tertiary children's hospital (The Hospital for Sick Children, Toronto, Canada). Referrals come from a variety of sources, including family physicians, primary care pediatricians, and pediatric subspecialists. STOMP uses a family-centered approach and is delivered by an interdisciplinary team of physicians, nurse practitioners, psychologists, dietitians, social work, exercise therapy, and physiotherapy. The Research Ethics Board at The Hospital for Sick Children approved this study. The RCT component was registered at clinicaltrials.gov.

### Participants

We included young children with obesity (age ≥1 year and <6 years) and their parent/primary caregiver [pilot RCT and parent focus groups], plus HCPs who delivered the STOMP-EY intervention [HCP interviews].

Obesity was defined as BMI ≥97th percentile for age and sex based on World Health Organization (WHO) growth reference charts at the time they were referred to the obesity management program.^18^ This definition (corresponding to those of the WHO for children ≥5 years^19^) was selected to be consistent across age groups and to correspond to clinical referral criteria.

### Intervention

**Figure 1.**
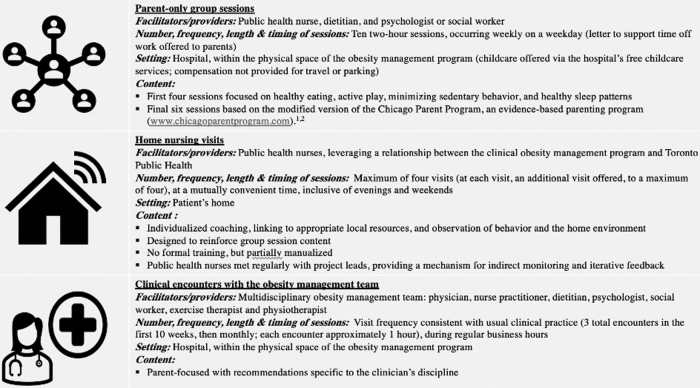
Core components of the STOMP-EY intervention. STOMP-EY, SickKids Team Obesity Management Program-Early Years.

Developed by STOMP members in conjunction with Toronto Public Health (including C.S.B., E.D., J.H., A.T., M.K.), STOMP-EY consisted of three core components delivered over 6 months: parent-only group sessions, home nursing visits, and clinical encounters with the obesity management team (Fig. 1). Usual clinical care consisted of clinical encounters with the multidisciplinary team, with the same frequency as the intervention group.

HCPs delivering parent-only group sessions had been trained in delivering group-based interventions and group facilitation with this age group. Public health nurses were embedded within the obesity management team and had specific training in parenting support. Parent-only group sessions and home nursing visits were manualized. In home visits, public health nurses reinforced principles from group sessions, which they cofacilitated, using a checklist to maintain fidelity.

#### Assessing Intervention Feasibility—Pilot RCT

##### Eligibility and recruitment

For RCT eligibility, the parent/primary caregiver had to be fluent in written and spoken English. We excluded children with Prader-Willi syndrome or severe developmental delay, and families residing outside the public health unit catchment area. Recruitment occurred from October 2017 to July 2019. A research coordinator (S.M.O.) screened families referred to the obesity management clinic and sent written information to those who were eligible. At the first clinical encounter, the research coordinator met with families to answer questions and obtain signed consent (or to ask about participation, if they could not be reached prior). After three unsuccessful contact attempts, it was assumed that a family was not interested in participating.

##### Sample size

Sample size was not formally calculated since this pilot RCT was not designed to provide a definitive estimate of treatment effect.^20^ Since our initial intention was to include pilot data as part of a large-scale RCT, we sought to include 42 parent-child dyads (*n* = 38 plus 10% attrition), based on recommendations for internal pilot studies.^21,22^

##### Randomization

Participants were randomized to the STOMP-EY intervention or to usual clinical care. Randomization occurred using a 1:1 allocation ratio, a computer-generated random allocation sequence, and opaque, sealed envelopes. After 6 months, STOMP-EY was offered to the usual care group.

##### Data collection

Study enrollment, intervention allocation, and follow-up were monitored to assess intervention feasibility, defined as our ability to recruit, consent, and retain participants.^23^ We also sought to determine feasibility of collecting and analyzing an *a priori*-defined clinical outcome: change in age- and sex-standardized BMI z-score (zBMI) 6 months postintervention start, or from baseline measurements for those randomized to usual clinical care. zBMI was determined using WHO growth standards^18,24^ a trained research assistant measured height (length for children <2 years) and weight using standardized anthropometric protocols.^25^

##### Analysis

Descriptive statistics (*e.g.*, medians, percentages) are reported.

#### Assessing Intervention Acceptability—Focus Groups with Parents

##### Eligibility and recruitment

We used purposeful sampling to recruit participants, who were invited to participate via telephone or email. Study eligibility required the parent/primary caregiver to be fluent in English and have internet access. To maximize the number of parents eligible to participate, and to capture a variety of parent perspectives, pilot RCT participation was not a prerequisite for focus group participation. Parents not enrolled in the RCT were familiar with STOMP-EY via the obesity management program (and may have received intervention components outside the pilot RCT).

##### Focus group methodology

To explore intervention acceptability—stakeholders' perceptions that the intervention is “agreeable, palatable, or satisfactory”^23^—focus groups (duration: ∼60 minutes) were conducted in April 2019 (*n* = 5 parents) and February 2020 (*n* = 3 parents). In total, three parents participated in the pilot RCT. Informed by Deverka et al.'s framework for effective engagement, a semistructured interview guide provided a framework for discussions that were facilitated by a research coordinator (S.M.O.) and qualitative researcher (E.S.).^26^ Participation occurred via Zoom, a secure online video conference platform (https://zoom.us/). Focus groups were audio recorded and transcribed verbatim.

##### Analysis

Focus group transcripts were analyzed thematically.^27^ Two investigators (S.M.O., R.D.) generated codes independently for all focus transcript data, and subsequently met to discuss and finalize codes. Disagreements were resolved by consensus. Codes that achieved consensus were organized and combined to form overarching themes that were further refined by checking for consistency between the data and identified codes and themes.

#### Examining Contextual Factors Impacting Intervention Implementation—Interviews with HCPs

##### Eligibility and recruitment

We recruited a purposive sample of 10 HCPs involved in STOMP-EY delivery.

##### Interview methodology

To examine contextual factors affecting intervention implementation, semistructured, 1-on-1 HCP interviews were conducted between November 2019 and March 2020, guided by the Consolidated Framework for Implementation Research (CFIR).^28^ Interviews were conducted by a single interviewer (E.S.), recorded, and transcribed verbatim. Each interview was ∼1 hour in length.

##### Analysis

The CFIR specifies constructs that may influence implementation (either positively or negatively), organized into five domains: intervention characteristics, inner setting, outer setting, characteristics of individuals involved, and implementation process.^28^ Guided by the CFIR, HCP interviews were coded by two coders [S.M.O. (all transcripts); R.D. (40% of transcripts)] and analyzed deductively using MAXQDA software. Based on coded HCP interviews, each CFIR construct was rated as to whether it was salient to STOMP-EY implementation and, if salient, whether it influenced implementation positively or negatively.

## Results

### Assessing Intervention Feasibility—Pilot RCT

**Figure 2.**
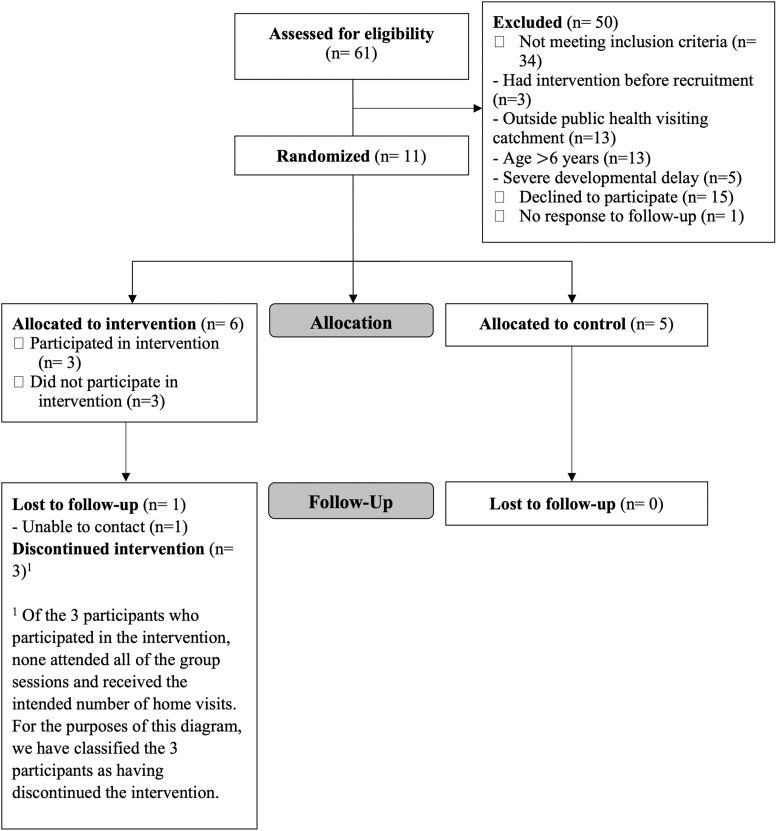
CONSORT flow diagram for the pilot randomized controlled trial of the STOMP-EY intervention. CONSORT, Consolidated Standards of Reporting Trials.

Participant eligibility, randomization, and follow-up are shown in Figure 2. Over 21 months, 61 parent-child dyads were screened for eligibility. Only 27 parent-child dyads met criteria and 11 were enrolled: 6 randomized to the intervention group and 5 to the usual care group. Most dyads that we screened were not eligible (34/61). The most common reasons for ineligibility were the child's age (too old; *n* = 13) and residence outside the public health unit catchment area (*n* = 13).

The consent rate was 40.7% (11/27). Baseline characteristics of participating children are shown in Table 1, with additional measurements pertaining to the study sample described in Supplementary Appendix SA1.

**Table 1.** Characteristics of Participating Children at Baseline

	Participating children (*n* = 11)
Sociodemographic characteristics	
Sex [No (%)]	
Male	5 (45.5)
Female	6 (54.5)
Age, years [median (range)]	4.6 (1.1, 7.2)
Anthropometric and physical measurements	
Height z-Score [median (range)]	1.41 (−0.21, 5.06)
Weight z-Score [median (range)]	4.59 (1.83, 11.7)
zBMI [median (range)]	5.61 (1.82,^a^ 11.2^b^)

^a^ One participant met criteria for inclusion based on a BMI ≥97th percentile (≅zBMI of 2) at the time of referral, but had a zBMI less than the 97th percentile at the time of baseline measurements.

^b^ zBMI of 11.2 was reviewed and was confirmed to be correct.

BMI, body mass index; zBMI, BMI z-score.

Among the six participants randomized to the intervention, STOMP-EY participation in the core components was variable. The number of group sessions attended, out of 10, ranged from 0 (3 participants) to 7 (1 participant). Uptake of home visits ranged from 0 (3 participants) to 2 (1 participant). One protocol violation was identified. The parent of a 7-year-old child who was ineligible, based on age, participated in STOMP-EY per randomization. Enrollment ceased once challenges around recruitment and participation became clear. Data pertaining to change in zBMI were collected for 10 of 11 participants (90.9%). Given the low number of participants and the low rate of intervention participation, variance around zBMI change (to inform a definitive RCT) could not be reliably estimated.

### Assessing Intervention Acceptability—Focus Groups with Parents

Themes that emerged in the two focus groups were consistent and are described below with illustrative quotes in Table 2.

**Table 2.** Themes Identified in Qualitative Analysis of Parent Focus Group Data, with Illustrative Quotes

Theme	Illustrative quote
Unclear and insufficient information at referral	“I was going with the assumption that this was just a blood test, I didn't understand that there was a whole program attached to it”
	“I just thought it was a dietitian, but I didn't know that it had more than just a dietician, so there was a social worker, a pediatrician, there was physical education person, and all those things…”
	“I actually thought it would be medicinal intervention, but I feel it didn't happen […] I kind of thought maybe there is some medicine to reduce appetite so he doesn't want to eat so much.”
Limited fit between intervention and patient needs	“My son also, he has a genetic deletion, like a genetic mutation, but he's not obese, he has hyperphagia.”
	“We were very skeptical because the issue with my son wasn't healthy eating or not healthy eating, so we were being put through a program […] it wasn't something that we necessarily needed and wanted to spend our time learning about.”
	“If you know what the families' needs are, I think you can tailor it to that, but the whole focus of the group is really about healthy eating and obesity and how you can better get your kids on a schedule, or a lot of it was basic parenting but that wasn't really what any of us thought we were in the program for.”
Parents' gains from participating in the intervention.	“It's a really good support team. And […] the nurses were great too with the home visits, they are always offering support, they are always calling to check in if everything is ok…”
	“It was great, I learned a lot through the whole 1 year and a half… I'm sharing some of the things I learned to my friends as well.”
Limited value and feasibility of group sessions	“…it just it didn't feel like a good use of time given all the arrangements I had to make to get there.”
	“I would make the time for it if I thought it was worthwhile and I thought we were benefiting.”

#### Unclear and insufficient program information at referral

Parents reported receiving very little information about the intervention from their referring physician, which limited their understanding of the intervention. Parents talked about feeling relief and hope when referred to an obesity management program, but most did not know what STOMP-EY entailed. This influenced parents' expectations and, in some cases, led to misrepresentation of what the intervention offered and required of them.

#### Limited fit between intervention and patient needs

The children of participating parents represented a heterogenous group with respect to diagnoses and treatment priority. Some children had complex medical issues such that weight management was not a priority for some families. The limited fit between the intervention and patient needs had implications for parent engagement and, ultimately, the perceived value of the intervention. Several parents talked about the need to tailor program content and recommendations to the unique needs of their children.

#### Parents' gains from participating in the intervention

Overall, there was consistency among parents with respect to the perceived value of the intervention, especially for clinical encounters and home visits. Parents appreciated the interdisciplinary nature of the intervention and the team's expertise and compassion. Those who participated in home visits felt that these relieved some stresses of daily life and helped with applying knowledge gained in the intervention. The core teachings of the intervention were perceived to lay a foundation for healthy living.

#### Limited value and feasibility of group sessions

Some parents perceived the group sessions as unfeasible and ineffective for various reasons: group engagement was hindered by limited and inconsistent parental attendance, decreasing engagement; difficulties committing to 10 weeks of sessions because of timing and disruptions to parents' schedules; and a feeling of being “talked at” rather than “talked to.” Parking costs and travel were also noted barriers. Most parents requested that their availability be considered in group scheduling. Some reported that their attendance was influenced by the perceived value of the intervention.

### Examining Contextual Factors Impacting STOMP Implementation—Interviews with HCPs

Salience and influence of the CFIR constructs on STOMP-EY implementation, based on HCP interviews, are presented in Table 3. Numerous constructs were rated as salient and positively influencing implementation (*e.g.*, evidence strength and quality, networks and communications, culture, self-efficacy). Conversely, numerous constructs were deemed to have negatively influenced STOMP-EY implementation. These negative influences were related to the characteristics of the intervention (*e.g.*, complexity), the inner setting of the intervention (*e.g.*, leadership engagement, available resources, access to knowledge, and information), and the process of implementation (*e.g.*, intervention participants, executing the implementation according to plan). The adaptability of the intervention was acknowledged by HCPs, who expressed that STOMP-EY would benefit from additional resources to support delivery, formal training for providers, and increased leadership engagement.

**Table 3.** Contextual Factors Affecting Implementation of the SickKids Team Obesity Management Program–Early Years Intervention Using the Consolidated Framework for Implementation Research, Based on Health Care Provider Interviews (*n* = 10)

Domains and constructs of CFIR	Salience (salient/did not manifest)	Influence on implementation (positive/negative/mixed)	Summary statement
(I) Intervention characteristics			
Intervention source	Did not manifest	NA	NA
Evidence strength and quality	Salient	Positive	HCPs were aware of the body of research that informed STOMP-EY and that intervention-related decisions were consistently based on best practices.
Relative advantage	Salient	Positive	STOMP-EY was perceived as “being in a different league, a good league,” and more effective than other existing interventions because of its team-based, comprehensive, holistic, multidisciplinary approach.
Adaptability	Salient	Positive	HCPs valued the adaptability of STOMP-EY with respect to timing of delivery, replacing the home visits with phone calls (or “coaching calls”) to solve barriers-related home visits, and personalizing parent plans.
Trialability	Did not manifest	NA	NA
Complexity	Salient	Negative	HCPs acknowledged the high coordination demands in delivering STOMP-EY, and the many components related to the multidisciplinary nature of the intervention.
Design quality and packaging	Salient	Mixed	HCPs appreciated the materials supporting the intervention (curriculum manual) but several found the intervention components related to parent communication repetitive and clumsy, the checking of the cupboards during home visits without prior notice to parents deceptive, and resources for parents too fragmented.
Cost	Salient	Mixed	HCPs commented on the free nature of the intervention for parents; some assumed that the cost of running STOMP-EY was high and, with low parental engagement, there was perhaps limited return on investment and sustainability.
(II) Outer setting			
Patient needs and resources	Salient	Positive	HCPs perceived STOMP-EY as being “client-centered” with patients' needs and feedback consistently informing the delivery of the intervention.
Cosmopolitanism	Salient	Positive	HCPs talked about feeling networked with external organizations, and spoke of the positive impact of knowing what others do, sharing best practices, identifying community resources, and being connected through various channels.
Peer pressure	Did not manifest	NA	NA
External policies and incentives	Salient	Mixed	Although HCPs were generally aware of the need for obesity interventions from various strategic plans, external policies, and incentives were not specific or actionable enough to be perceived as a strong facilitator of the intervention.
(III) Inner setting			
Structural characteristics	Salient	Positive	HCPs perceived the age and maturity of the organization as having a positive influence on STOMP-EY implementation.
Networks and communications	Salient	Positive	HCPs characterized the communication protocols as being effective and acknowledged the many opportunities for communication through medical rounds or smaller groups, and the team being very good at “cutting to the chase.”
Culture	Salient	Positive	Collaboration, support, quality, and patient-focus were consistently noted as values underlying the intervention and the organizational culture was seen as highly inclusive, supportive and evidence-based.
Implementation climate			
Tension for change	Did not manifest	NA	NA
Compatibility	Salient	Mixed	HCPs found that STOMP-EY fit existing workflows and structures, but some noted that components (*e.g.,* home visits) were too short or difficult to schedule without having very long days or requiring overtime. In addition, some patients were perceived as being complex and not being the best fit for STOMP-EY.
Relative priority	Salient	Mixed	STOMP-EY implementation was regarded as important and meeting the needs of the patients, but limited time and coordination reduced, at times, its priority status.
Organizational incentives and rewards	Salient	Mixed	Overall HCPs talked about having limited formal organizational recognition and rewards for being involved with STOMP-EY, although informally, in the smaller team, the opposite was true.
Goals and feedback	Salient	Mixed	Although HCPs acknowledged the presence of goals and feedback as part of STOMP-EY implementation, the general feeling was that it was too informal and would have benefitted from being more explicit and structured.
Learning climate	Salient	Positive	HCPs talked about feeling valued, appreciated by parents and having a collaborative, supportive, and enthusiastic work environment where feedback was often sought from team members and carefully considered.
Readiness for implementation			
Leadership engagement	Salient	Negative	Leadership engagement was generally perceived as limited and a missed opportunity to support STOMP-EY implementation.
Available resources	Salient	Negative	HCPs spoke of the need for more formal and cohesive training, additional resources and a coordinator role to support STOMP-EY delivery. HCPs also talked about needing more time for STOMP-EY implementation.
Access to knowledge and information	Salient	Negative	Overall, HCPs spoke of having limited understanding of the research goals and the type of observations necessary to support STOMP-EY. In addition, HCPs talked about the need for more information to help them move from generic to more individualized/tailored support for parents.
(IV) Characteristics of individuals			
Knowledge and beliefs about the intervention	Salient	Positive	HCPs regarded STOMP-EY positively because of its unique, comprehensive, multidisciplinary, evidence-based approach and the perceived positive effects for families.
Self-efficacy	Yes	Positive	Self-efficacy in delivering STOMP-EY was consistently noted as having a facilitating effect; having prior experience in working with children with special needs was seen as additionally contributing to HCPs feeling competent in delivering STOMP-EY.
Individual stage of change	Did not manifest	NA	NA
Individual identification with organization	Did not manifest	NA	NA
Other personal attributes	Did not manifest	NA	NA
(V) Process			
Planning	Did not manifest	NA	NA
Engaging			
Opinion leaders	Did not manifest	NA	NA
External change agents	Did not manifest	NA	NA
Formally appointed internal implementation leaders	Salient	Positive	Managerial support was regarded positively and as having a facilitating effect on STOMP-EY implementation.
Champions	Salient	Positive	The presence of enthusiastic, knowledgeable, and committed individuals was valued and perceived as a strong facilitator for STOMP.
Intervention participants^a^	Salient	Negative	Low attendance (due to distance, time, travel); parents having limited to no understanding at referral of the intervention; parents' beliefs about the intervention and causes for their children's obesity; and existing comorbidities were some of the reasons noted for low engagement with STOMP-EY.
Executing	Salient	Negative	HCPs identified low group attendance as a contributor to difficulties with execution. Low group attendance was felt to negatively affect the dynamics, impact and quality of group discussions and interactions. HCPs also spoke of the need for fidelity measures for the home visit component of STOMP-EY.
Reflecting and evaluating	Salient	Positive	Overall, HCPs valued team meetings and other opportunities to reflect on STOMP-EY as a group, discuss progress and make informed adjustments.

^a^ The construct “intervention participants” is not part of the original CFIR framework; it was added by the research team to examine an aspect of the implementation that is related to the recipients of the intervention and their role in implementation, which is not captured by the framework.

CFIR, Consolidated Framework for Implementation Research; HCP, health care provider; NA, not applicable; STOMP-EY, SickKids Team Obesity Management Program-Early Years.

The complexity of the patient population was also acknowledged; HCPs perceived that increased access to resources would be helpful in providing more individualized support. Specific to parent-only group sessions, HCPs identified low attendance and engagement as challenges that negatively impacted group dynamics and the quality of group discussions. With respect to home visits, HCPs identified a need to ensure fidelity in delivery.

## Discussion

Addressing a gap in evidence-informed practice, we developed and implemented the STOMP-EY intervention for young children with obesity and their families. We encountered challenges with recruitment and participation in a pilot RCT of STOMP-EY, indicating a lack of feasibility in our setting: eligibility rate was <50%, consent rate was <50%, and half of those randomized to STOMP-EY did not participate in group sessions or home visits. Qualitative data from parents and HCPs revealed important insights on intervention acceptability and contextual factors affecting implementation. Taken together, our findings highlight important but modifiable barriers to successful implementation of STOMP-EY. These barriers were a low number of eligible participants, insufficient information provided to parents about the intervention at referral, lack of relative priority and perceived patient need, lack of tailoring to individual patient needs, poor parental motivation to engage in group sessions, and challenges related to scheduling and delivery of group sessions.

The number of potential participants screened for eligibility was low, and the proportion of ineligibility in those screened was high. The obesity management clinic from which we recruited participants may have contributed to the low number of eligible participants. Relatively few new assessments were completed monthly in the eligible age group, and many patients were from outside the immediate geographic area of the tertiary care hospital (rendering them ineligible for the intervention because they lived outside the public health unit catchment). Recruitment may have been more successful from community practices within the public health catchment area. Previous studies have demonstrated relatively high recruitment, engagement, and retention with weight management interventions in primary care and/or community settings.^16,29,30^

Given that children access primary care over 11 times in the first few years of life in our locale, primary care may be a more appropriate setting to implement and test interventions for young children with obesity. Alternatively, tertiary care-public health partnerships could be expanded to better reflect the demographics of the clinical population; however, doing so in advance of demonstrating intervention effectiveness is not recommended, as this expansion would require substantial resources.

As identified by both parents and HCPs, there was insufficient information provided to parents about the intervention at referral. In previous work, Smith et al. noted that, when contacted about obesity management program intake, many families were unaware of the reason for referral, or were uninterested in participating in the type of lifestyle intervention to which they had been referred.^31^ Failure to meet family expectations has been previously identified as an important factor in attrition from pediatric weight management programs,^32–34^ highlighting the importance of communicating about an intervention early in the engagement pathway.^34,35^ Early review of STOMP-EY's approach would allow parents to decide whether the program aligns with their expectations and perceived needs.

Written information on the intervention, as part of the electronic referral process, could partially address this issue. Although more resource intensive, an interactive information session for families could be implemented to ensure that the intervention has been directly, accurately, and thoroughly described.^31^ Whiteboard-style videos, including visual depiction of the intervention, might also be useful; such videos have shown promise in increasing parents' and HCPs' self-efficacy in communicating about weight and weight management.^36,37^ We hypothesize that adequate parental understanding of the intervention would facilitate STOMP-EY implementation by increasing engagement and decreasing attrition.

In the context of other medical issues, weight management's lack of relative priority emerged as a theme in parent focus groups. HCPs also perceived medical comorbidities as a barrier to successful STOMP-EY implementation. Family motivation is recognized as an important determinant of engaging in treatment and successfully managing pediatric obesity.^38^ We suggest that parents should be empowered to decide not only whether to participate in STOMP-EY, but also when to participate in STOMP-EY. An explicit option to revisit enrollment at a future time might facilitate participation and engagement.

Parents identified a lack of adapting program content to individual patient needs. HCPs also expressed wanting to move toward more individualized support for families. The need to tailor weight management interventions to the individual needs of each family has been previously described, and individualized care that is responsive to cultural and socioeconomic diversity, and children's needs is explicitly addressed in recommendations to improve health services for managing pediatric obesity in Canada.^39,40^ We propose increasing the interactivity of group sessions and encouraging participants to share their unique challenges and needs. Such sharing of individual experiences might be beneficial to the broader group and identify previously unrecognized commonalities.^41,42^ Any individualized needs that cannot be effectively addressed in the group setting could be redirected to home visits and individual clinical encounters.

We experienced low participation in group sessions, and low parental engagement in group sessions. In their interviews, HCPs reflected on some of these challenges around group dynamics and their negative influence on STOMP-EY. As social interaction and support have been identified as key drivers of ongoing attendance at community-based lifestyle interventions, we propose increasing the interactive component of sessions.^41^ We also propose incorporating components of motivational interviewing (MI).^43^ Although MI has largely been applied and studied in individual encounters, it has been used in group settings, predominantly in the field of substance use and addictions.^44–46^ Clinical encounters and home nursing visits would allow for more individualized application of MI techniques.

Challenges related to scheduling and delivery of group sessions could be addressed through a more collaborative approaching to scheduling (*i.e.*, working with participants to find a maximally convenient time among the group). Transitioning to an online/virtual format would address some practical challenges (*e.g.*, parking costs, travel time) and might therefore increase accessibility of group sessions; however, it has been suggested that web-based interventions may actually exacerbate inequities across sociodemographic groups and the “digital divide.”^47,48^ To our knowledge, the effectiveness of virtual small group sessions in pediatric weight management has not been studied. In adults, recent studies suggest that lifestyle interventions delivered via videoconference are at least as effective as standard interventions^49–51^; however, intervention or participant characteristics that contribute to the effectiveness of virtual small group sessions are not well understood. Changing STOMP-EY group sessions to an online/virtual format would therefore raise important considerations related to implementation, effectiveness, and equity.

## Limitations

First, we did not require pilot RCT participation for parent focus group eligibility. We believe that including a broader sample of parents provided valuable insights into the acceptability of STOMP-EY; however, we acknowledge that the intervention may have been perceived differently based on RCT enrollment. Themes emerging were not necessarily informed by direct experience of all intervention components via trial participation.

Second, we did not formally assess fidelity of STOMP-EY implementation. Assessing whether the intervention was implemented as intended would have allowed for a more comprehensive evaluation of implementation, and will be important for future iterations of the intervention.

## Conclusions

We encountered challenges related to feasibility and acceptability of the STOMP-EY intervention and identified contextual factors affecting implementation in our setting that might have been avoided through content codevelopment and a more explicit implementation approach to delivery. Identified barriers to successful implementation are, however, modifiable and can inform the next iteration of the intervention. Our findings also have broader implications for the development and delivery of interventions for young children with obesity. We suggest that future interventions acknowledge the importance of the intervention setting, inform parents about the intervention early in the engagement process, empower families to decide whether and when to participate, offer individualized support tailored to the diverse needs of families, consider opportunities to increase engagement in group sessions, and address how best to deliver intervention components.

## Supplementary Material

Supplemental data

Supplemental data
