# Supplementary material for: Managing Obesity in Young Children: A Multiple Methods Study Assessing Feasibility, Acceptability, and Implementation of a Multicomponent, Family-Based Intervention
Source: Child Obes. 2022 Aug 29;18(6):409–21. doi: 10.1089/chi.2021.0221 (PMC9492792; doi:10.1089/chi.2021.0221)
Supplement: Supplemental data [file Supp_Data.docx]

**Appendix.** Additional measurements and characteristics of the study sample in the pilot randomized controlled trial (RCT).

Additional measurements in the pilot RCT included sociodemographic characteristics, blood pressure, laboratory testing, nutrition, sleep habits, measures of child temperament, child mental health, family psychosocial health, and parenting.

Sociodemographic characteristics, food security, nutrition and sleep habits were obtained via chart review based on routinely-collected information in the clinical obesity management program.

Child temperament was measured using the 36-item very short form (VSF) of the Children’s Behavior Questionnaire (CBQ; 3 years and older) or Early Childhood Behavior Questionnaire (ECBQ; 2-3 years), which assess reactive and self-regulative temperamental behavior patterns in young children across three domains: surgency, negative affect, and effortful control. Items are assessed using a 7-point Likert scale ranging from 1 (extremely untrue of your child) to 7 (extremely true of child), and a higher score indicates greater symptomology.^1^

Child mental health was assessed using the Strengths and Difficulties Questionnaire (SDQ), which consists of 25 items. The SDQ Total Difficulties Score (TDS) can be calculated as the sum of the scores for the emotional symptoms; conduct problems, hyperactivity-inattention, and peer problems subscales. Each item has to be scored on a 3-point scale with 0 = ‘not true’, 1 = ‘somewhat true’ and 2 = ‘certainly true’. A higher score indicates more emotional and behavioral problems. Cutoffs for categorizing the TDS subscale into “normal, borderline, and abnormal” have been proposed.^2^

Family psychosocial health was assessed using the Depression, Anxiety, Stress Scale (DASS-21) and Parenting Stress Index Short Form (PSI-SF). DASS-21 is parent-completed and contains 21-items, prompting respondents to evaluate their experiences of depression, anxiety, and stress over the past week. Each item has a four-point Likert scale, ranging from 0 (did not apply to me at all) to 3 (applied to me very much/most of the time), with higher scores indicating greater symptomology. Cutoffs for categorizing each subscale into “normal, mild, moderate, severe, and extremely severe” have been proposed.^3^ The PSI-SF is a 36-item, parent-completed form designed to identify potentially dysfunctional parent-child systems and includes three scales: parental distress, difficult child characteristics, and dysfunctional parent-child interaction. Cutoffs for categorizing each subscale into “typical, high, and clinically significant” have been proposed.^4^

Parenting was captured through the Parenting Scale (PS), which is a 30-item measure to assess parental discipline strategies over three domains: laxness, over reactivity, and verbosity. Parent respondents rate their likelihood of using a given discipline strategy in response to a child’s misbehaviour. Each item has a 7-point scale that is anchored by one effective and one ineffective strategy, representing a score of 1 and 7 respectively.^5^

Blood pressure (systolic and diastolic) was obtained by trained research assistants using an appropriately sized cuff following guidelines from the National High Blood Pressure Education Program.^6^

Laboratory testing required 9-12 mL of blood, which was collected by a trained health professional experienced with pediatric blood collection. Analysis was carried out at the Pediatric Medicine laboratory using a number of different instruments.

**Appendix – Table 1.** Characteristics of the pilot randomized controlled trial (RCT) study sample.

|  | | | | **Participating children (n=11)** |  |
| --- | --- | --- | --- | --- | --- |
| ***Sociodemographic characteristics & food security*** | | | |  |  |
| Household income [No (%)] | | | |  |  |
|  | *<$50,00* | | | 3 (27.3) |  |
|  | *$50,000-$79,999* | | | 3 (27.3) |  |
|  | *≥$80,000* | | | 2 (18.2) |  |
|  | *Missing* | | | 3 (27.3) |  |
| Difficulty buying healthy food within the month [No (%)] | | | |  |  |
|  | *Never* | | | 5 (45.5) |  |
|  | *Sometimes* | | | 4 (36.4) |  |
|  | *Often* | | | 1 (9.1) |  |
|  | *Missing* | | | 1 (9.1) |  |
| Accessing food banks within the month [No (%)] | | | |  |  |
|  | *Never* | | | 7 (63.6) |  |
|  | *Sometimes* | | | 3 (27.3) |  |
|  | *Often* | | | 0 (0) |  |
|  | *Missing* | | | 1 (9.1) |  |
| ***Nutrition*** | | | |  |  |
| Ever breastfed [No (%)] | | | |  |  |
|  | | *Yes* | | 7 (63.6) |  |
|  | | *No* | | 0 (0) |  |
|  | | *Missing* | | 4 (36.4) |  |
| At least 1 serving of fruit per day [No (%)] | | | |  |  |
|  | | *Yes* | | 8 (72.7) |  |
|  | | *No* | | 2 (18.2) |  |
|  | | *Missing* | | 1 (9.1) |  |
| At least 1 serving of vegetables per day [No (%)] | | | |  |  |
|  | | *Yes* | | 6 (54.5) |  |
|  | | *No* | | 2 (18.2) |  |
|  | | *Missing* | | 3 (27.3) |  |
| At least 1 meal in front of the TV per day [No (%)] | | | |  |  |
|  | | *Yes* | | 5 (45.5) |  |
|  | | *No* | | 2 (18.2) |  |
|  | | *Missing* | | 4 (36.4) |  |
| Picky eating, per parents’ report [No (%)] | | | |  |  |
|  | | *Yes* | | 4 (36.4) |  |
|  | | *No* | | 4 (36.4) |  |
|  | | *Missing* | | 3 (27.3) |  |
| ***Sleep*** | | | |  |  |
| Weekday sleep, hours per night (median, standard deviation)^2^ | | | | 10.38 (9.75,12) |  |
| ***Child temperament and family psychosocial health*** | | | |  |  |
| Child temperament score, out of 7 [median (range)] | | | |  |  |
| *Surgency* | | | | 4.5 (4.17,5.50) |  |
| *Negative Affect* | | | | 4.17 (2.82,5.67) |  |
| *Effortful Control* | | | | 5.67 (3.25,6.58) |  |
| Parent mental health, as measured using DASS-21 | | | |  |  |
| *Stress* [No (%)] | | | |  |  |
| *Normal/mild* | | | | 9 (81.8) |  |
| *Moderate/Severe/Extremely severe* | | | | 2 (18.2) |  |
| *Anxiety* [No (%)] | | | |  |  |
| *Normal/mild* | | | | 7 (63.6) |  |
| *Moderate/Severe/Extremely severe* | | | | 4 (36.4) |  |
| *Depression* [No (%)] | | | |  |  |
| *Normal* | | | | 7 (63.6) |  |
| *Moderate/Severe/Extremely severe* | | | | 4 (36.4) |  |
| Parenting stress, as measured using PSI-4 SF | | | |  |  |
| *Parental Distress* | | | |  |  |
| *Typical/high (<90^th^ percentile)* | | | | 8 (72.7) |  |
| *Clinically Significant (>90^th^ percentile)* | | | | 3 (27.3) |  |
| *Parent-Child Dysfunctional Interaction* | | | |  |  |
| *Typical/high (<90^th^ percentile)* | | | | 10 (90.9) |  |
| *Clinically Significant (>90^th^ percentile)* | | | | 1 (9.1) |  |
| *Difficult Child* | | | |  |  |
| *Typical/high (<90^th^ percentile)* | | | | 11 (100) |  |
| *Clinically Significant (>90^th^ percentile)* | | | | 0 (0) |  |
| *Total Stress* | | | |  |  |
| *Typical/high (<90^th^ percentile)* | | | | 10 (90.9) |  |
| *Clinically Significant (>90^th^ percentile)* | | | | 1 (9.1) |  |
| Child Mental Health | | | |  |  |
| *SDQ Total Score* | | | |  |  |
| *Normal/Borderline* | | | | 2 (18.2) |  |
| *Abnormal* | | | | 5 (45.5) |  |
| *Missing* | | | | 4 (36.4) |  |
| Parenting scale, out of 7 [median (range)]^1^ | | | |  |  |
| *Laxness* | | | | 3.27 (1.73,5.09) |  |
| *Over-reactivity* | | | | 2.39 (1.56,4.67) |  |
| *Hostility* | | | | 4.29 (3.43,6.00) |  |
| ***Physical measurements*** | | | |  |  |
| Blood pressure percentile [median (range)]^1^ | | | |  |  |
| *Systolic* | | | | 30.5 (6,80) |  |
| *Diastolic* | | | | 67 (27,88) |  |
| ***Biochemical measurements*** | | | |  |  |
| Total Cholesterol, mmol/L [median (range)] | | | | 3.93 (2.87,6.24) |  |
| Triglycerides, mmol/L [median (range)] | | | | 1.0 (0.38,3.71) |  |
| HDL, mmol/L [median (range)] | | | | 1.08 (0.71,1.37) |  |
| LDL, mmol/L [median (range)] | | | | 2.45 (1.49,3.65) |  |
| Fasting glucose, mmol/L [median (range)] | | | | 4.9 (4.4,5.6) |  |
| Hemoglobin A1C, % [median (range)] | | | | 5.0 (4.1,5.2) |  |
| CRP, mg/L [median (range)] | | | | 2.3 (0.3,94.3) |  |
| Ferritin, ug/L [median (range)] | | | | 31.6 (3.2,70.5) |  |
| Hemoglobin, g/L [median (range)] | | | | 116 (92,136) |  |
|  | | |  |  |  |

^1^ N=10

^2^ N=8

**Appendix - References**

1. [Putnam, S. P., & Rothbart, M. K. (2006). Development of Short and Very Short forms of the Children’s Behavior Questionnaire. Journal of Personality Assessment, 87 (1), 103-113.](http://research.bowdoin.edu/rothbart-temperament-questionnaires/files/2016/09/cbq_short_form_paper.pdf)
2. Goodman A, Goodman R. Strengths and Difficulties Questionnaire scores and mental health in looked after children. Br J Psychiatry 2012;200:426-7.
3. Lovibond, S.H. & Lovibond, P.F. (1995). Manual for the Depression Anxiety & Stress Scales. (2nd Ed.)Sydney: Psychology Foundation.
4. Abidin RR. Parenting Stress Index: Professional manual, 3rd ed. Odessa, FL, USA: Psychological Assessment Resources; 1995.
5. Arnold DS, O’Leary SG, Wolff LS, Acker MM. The Parenting Scale: A measure of dysfunctional parenting in discipline situations. Psychological Assessment 1993;5(2):137-44.
6. The fourth report on the diagnosis, evaluation, and treatment of high blood pressure in children and adolescents. Pediatrics. 2004;114:555-576. <https://doi.org/10.1542/peds.114.2.S2.555>.)
