# Supplementary material for: Managing Obesity in Young Children: A Multiple Methods Study Assessing Feasibility, Acceptability, and Implementation of a Multicomponent, Family-Based Intervention
Source: Child Obes. 2022 Aug 29;18(6):409–21. doi: 10.1089/chi.2021.0221 (PMC9492792; doi:10.1089/chi.2021.0221)
Supplement: Supplemental data [file Supp_TableS1.docx]

**Appendix – Table 1.** Characteristics of the pilot randomized controlled trial (RCT) study sample.

|  | | | | **Participating children (n=11)** |  |
| --- | --- | --- | --- | --- | --- |
| ***Sociodemographic characteristics & food security*** | | | |  |  |
| Household income [No (%)] | | | |  |  |
|  | *<$50,00* | | | 3 (27.3) |  |
|  | *$50,000-$79,999* | | | 3 (27.3) |  |
|  | *≥$80,000* | | | 2 (18.2) |  |
|  | *Missing* | | | 3 (27.3) |  |
| Difficulty buying healthy food within the month [No (%)] | | | |  |  |
|  | *Never* | | | 5 (45.5) |  |
|  | *Sometimes* | | | 4 (36.4) |  |
|  | *Often* | | | 1 (9.1) |  |
|  | *Missing* | | | 1 (9.1) |  |
| Accessing food banks within the month [No (%)] | | | |  |  |
|  | *Never* | | | 7 (63.6) |  |
|  | *Sometimes* | | | 3 (27.3) |  |
|  | *Often* | | | 0 (0) |  |
|  | *Missing* | | | 1 (9.1) |  |
| ***Nutrition*** | | | |  |  |
| Ever breastfed [No (%)] | | | |  |  |
|  | | *Yes* | | 7 (63.6) |  |
|  | | *No* | | 0 (0) |  |
|  | | *Missing* | | 4 (36.4) |  |
| At least 1 serving of fruit per day [No (%)] | | | |  |  |
|  | | *Yes* | | 8 (72.7) |  |
|  | | *No* | | 2 (18.2) |  |
|  | | *Missing* | | 1 (9.1) |  |
| At least 1 serving of vegetables per day [No (%)] | | | |  |  |
|  | | *Yes* | | 6 (54.5) |  |
|  | | *No* | | 2 (18.2) |  |
|  | | *Missing* | | 3 (27.3) |  |
| At least 1 meal in front of the TV per day [No (%)] | | | |  |  |
|  | | *Yes* | | 5 (45.5) |  |
|  | | *No* | | 2 (18.2) |  |
|  | | *Missing* | | 4 (36.4) |  |
| Picky eating, per parents’ report [No (%)] | | | |  |  |
|  | | *Yes* | | 4 (36.4) |  |
|  | | *No* | | 4 (36.4) |  |
|  | | *Missing* | | 3 (27.3) |  |
| ***Sleep*** | | | |  |  |
| Weekday sleep, hours per night (median, standard deviation)^2^ | | | | 10.38 (9.75,12) |  |
| ***Child temperament and family psychosocial health*** | | | |  |  |
| Child temperament score, out of 7 [median (range)] | | | |  |  |
| *Surgency* | | | | 4.5 (4.17,5.50) |  |
| *Negative Affect* | | | | 4.17 (2.82,5.67) |  |
| *Effortful Control* | | | | 5.67 (3.25,6.58) |  |
| Parent mental health, as measured using DASS-21 | | | |  |  |
| *Stress* [No (%)] | | | |  |  |
| *Normal/mild* | | | | 9 (81.8) |  |
| *Moderate/Severe/Extremely severe* | | | | 2 (18.2) |  |
| *Anxiety* [No (%)] | | | |  |  |
| *Normal/mild* | | | | 7 (63.6) |  |
| *Moderate/Severe/Extremely severe* | | | | 4 (36.4) |  |
| *Depression* [No (%)] | | | |  |  |
| *Normal* | | | | 7 (63.6) |  |
| *Moderate/Severe/Extremely severe* | | | | 4 (36.4) |  |
| Parenting stress, as measured using PSI-4 SF | | | |  |  |
| *Parental Distress* | | | |  |  |
| *Typical/high (<90^th^ percentile)* | | | | 8 (72.7) |  |
| *Clinically Significant (>90^th^ percentile)* | | | | 3 (27.3) |  |
| *Parent-Child Dysfunctional Interaction* | | | |  |  |
| *Typical/high (<90^th^ percentile)* | | | | 10 (90.9) |  |
| *Clinically Significant (>90^th^ percentile)* | | | | 1 (9.1) |  |
| *Difficult Child* | | | |  |  |
| *Typical/high (<90^th^ percentile)* | | | | 11 (100) |  |
| *Clinically Significant (>90^th^ percentile)* | | | | 0 (0) |  |
| *Total Stress* | | | |  |  |
| *Typical/high (<90^th^ percentile)* | | | | 10 (90.9) |  |
| *Clinically Significant (>90^th^ percentile)* | | | | 1 (9.1) |  |
| Child Mental Health | | | |  |  |
| *SDQ Total Score* | | | |  |  |
| *Normal/Borderline* | | | | 2 (18.2) |  |
| *Abnormal* | | | | 5 (45.5) |  |
| *Missing* | | | | 4 (36.4) |  |
| Parenting scale, out of 7 [median (range)]^1^ | | | |  |  |
| *Laxness* | | | | 3.27 (1.73,5.09) |  |
| *Over-reactivity* | | | | 2.39 (1.56,4.67) |  |
| *Hostility* | | | | 4.29 (3.43,6.00) |  |
| ***Physical measurements*** | | | |  |  |
| Blood pressure percentile [median (range)]^1^ | | | |  |  |
| *Systolic* | | | | 30.5 (6,80) |  |
| *Diastolic* | | | | 67 (27,88) |  |
| ***Biochemical measurements*** | | | |  |  |
| Total Cholesterol, mmol/L [median (range)] | | | | 3.93 (2.87,6.24) |  |
| Triglycerides, mmol/L [median (range)] | | | | 1.0 (0.38,3.71) |  |
| HDL, mmol/L [median (range)] | | | | 1.08 (0.71,1.37) |  |
| LDL, mmol/L [median (range)] | | | | 2.45 (1.49,3.65) |  |
| Fasting glucose, mmol/L [median (range)] | | | | 4.9 (4.4,5.6) |  |
| Hemoglobin A1C, % [median (range)] | | | | 5.0 (4.1,5.2) |  |
| CRP, mg/L [median (range)] | | | | 2.3 (0.3,94.3) |  |
| Ferritin, ug/L [median (range)] | | | | 31.6 (3.2,70.5) |  |
| Hemoglobin, g/L [median (range)] | | | | 116 (92,136) |  |
|  | | |  |  |  |

^1^ N=10

^2^ N=8
